# Catalytic Properties of Two Complexes of chromium(III) and cobalt(II) with Nitrilotriacetate, Dipicolinate, and 4-Acetylpyridine

**DOI:** 10.3390/ma16093308

**Published:** 2023-04-23

**Authors:** Jacek Malinowski, Joanna Drzeżdżon, Katarzyna N. Jarzembska, Radosław Kamiński, Przemysław Rybiński, Barbara Gawdzik, Dagmara Jacewicz

**Affiliations:** 1 Faculty of Chemistry, University of Gdansk (Gdańsk), Wita Stwosza 63, 80-308 Gdansk, Poland; 2 Department of Chemistry, University of Warsaw, Żwirki i Wigury 101, 02-089 Warsaw, Poland; 3 Institute of Chemistry, Jan Kochanowski University, Uniwersytecka 7, 25-406 Kielce, Poland

**Keywords:** crystal structure, cobalt(II) and chromium(III) complexes, oligomerization, methylaluminoxane, 2-chloro-2-propen-1-ol

## Abstract

In this paper, a synthesis of two innovative coordination compounds, based on chromium(III) and cobalt(II) ions with N,O-donor ligands (nitrilotriacetate, dipicolinate) and 4-acetylpyridine, is reported. The obtained metal-organic compounds were structurally characterized using the single-crystal X-ray diffraction (XRD) method. The well-defined chromium(III) and cobalt(II) complexes were used as precatalysts in the oligomerization reaction of 2-chloro-2-propen-1-ol and 2-propen-1-ol with methylaluminoxane (MMAO) as an activator. The products of the oligomerization reaction were subjected to full physicochemical characteristics, i.e., time-of-flight mass spectrometry (MALDI-TOF-MS), TGA, and differential scanning calorimetry (DSC) methods. The catalytic activity of the precatalysts in both reactions was calculated and compared with other catalysts known in the literature.

## 1. Introduction

The most commonly used catalysts in the oligomerization of olefins are acids and transition metal coordination compounds. They are used in the industrial oligomerization processes of light olefins, which find applications in the production of fuels, lubricants, petrochemicals, and other chemicals [1,2,3,4].

Each catalyst produces or leads to the formation of an oligomer with a different microstructure and properties. The obtained materials may differ in thermal durability, for example [5,6,7,8,9]. For instance, nickel(II)-centre catalysts show the ability to carry out oligomerization reactions of linear butenes or propenes, which most often undergo chain dimerization reactions [10,11,12,13,14,15,16].

Precatalysts based on heavy-atom metal centers have been quite well studied and described in the last three decades. The structure of the precatalyst, the choice of activator or solvents, and the parameters for conducting the oligomerization process make it possible to obtain a product with a different structure or to finetune the catalytic activity of the precatalyst used [17,18,19,20,21,22,23,24,25,26,27,28,29,30]. Catalysts based on nickel or palladium cations are considered to have played an early role in the development of Ziegler–Natta catalysts. These catalysts are most often used in the initial stages of the oligomerization of olefins. The Shell Higher Olefin Process (SHOP), which yields linear alpha-olefins, uses a nickel catalyst that has phosphorus and oxygen donor atoms in its structure [31].

Metallocene and post-metallocene catalysts of early and further transition metals are built with a metal center and coordinated organic ligands. The most widely used ligands are compounds whose structure contains donor atoms such as P, O, N, and S. This is due to the fact that these atoms make it possible to design the surroundings of the central metal according to the needs and pros of the catalyst application [32].

An important discovery in the field of catalysts was published in 1998 by Gibson and Brookhart. Both showed that 2,6-bis(phenylino)pyridyl complexes of iron(II) and cobalt(II) exhibit high activity in the oligomerization and polymerization reactions of ethylene. The timing prompted other research groups to use complex compounds with different steric or electron properties. Specific changes in the electron or steric structure allow better control of the oligomerization or polymerization products of olefins [33].

Our work aimed to fill the research niche by designing the synthesis of potential precatalysts, based on chromium(III) and cobalt(II) ions, and their full physicochemical characterization. In-depth structural studies were made possible by growing single crystals of the cobalt(II) (**1**) and chromium(III) (**2**) coordination compounds. In addition, we checked the catalytic properties of (**1**) and (**2**) in the process of olefin oligomerization as well as the physicochemical characteristics of the obtained oligomers. The correlation of the results of the structural studies obtained for the (**1**) and (**2**) complex compounds with the outcomes of the catalytic and spectroscopic studies allows us to determine a few structure–property relationships, important to finetune selected physicochemical parameters.

## 2. Materials and Methods

The syntheses of newly obtained complex compounds:

**Synthesis of complex (1)**

To an aqueous solution of cobalt(II) chloride hexahydrate (2 mmol) was added an aqueous solution of nitrilotriacetic acid (2 mmol). The prepared solution was stirred on a magnetic stirrer under a reflux condenser for 2 h. The mixture was then heated for 3 h at 40 °C under atmospheric pressure. After about 4 months, fine pink crystals were obtained. The solution was concentrated and left for another week. Then the obtained compound was washed several times with ethanol and left to dry. Pink crystals of the complex compound were obtained.

**Synthesis of complex (2)**

The synthesis was started by dissolving 2,6-pyridinedicarboxylic acid (0.34 g) in 40 mL of ethanol. Then an aqueous solution of chromium(III) chloride was prepared by dissolving 0.54 g in 15 mL of water. The acid solution was combined with the chromium(III) salt solution. The resulting mixture was left on a magnetic stirrer for 4h. Then 220 µL of 4-acetylpyridine was added. After adding the last substrate, the mixture was refluxed at 50 °C. All the time the resulting mixture was on a magnetic stirrer. In the next step the mixture was heated under reflux for 5 h. Purple crystals of the complex compound were obtained.

**X-ray structural studies**

All single-crystal X-ray measurements were performed on a Rigaku Oxford Diffraction SuperNova instrument equipped with a microfocus copper X-ray source (Cu K_α_ radiation, λ = 1.54184 Å). During the measurements, crystals were maintained at 100 K with the use of an Oxford Cryosystems nitrogen gas-flow device. Unit-cell parameter determination and raw diffraction image processing were performed with the native diffractometer CRYSALISPRO software suite. Structures were solved using an intrinsic phasing method as implemented in the SHELXT program [34] and refined with the JANA package [35] within the independent atom model approximation. In the case of C-H and N-H bonds, the hydrogen atoms were placed geometrically (d_(C-H) = 0.96 Å, d_(N-H) = 0.87 Å). Oxygen-bound hydrogen atoms were refined with the O-H bond-distance restraints set to 0.82 Å. In all cases the riding model for the hydrogen thermal motion parameters was applied (U_iso^H = x∙U_eq^X where x = 1.2 for X = C, and x = 1.5 for X = O, N). In the case of (**2**) the proposed protonation of the 4-acetylpyridine nitrogen atom seems most reasonable regarding the charge balance of this system and provides the lowest 𝑅-factor. For more details see Supporting Information. CCDC 2116382 for (**1**) and CCDC 2116383 for (**2**) contain the supplementary crystallographic data for this paper. These data can be obtained free of charge from The Cambridge Crystallographic Data Centre via www.ccdc.cam.ac.uk/structures accessed on 10 April 2023 [36,37].

**IR analysis**

A Thermo Scientific Nicolet iS5 FTIR spectrometer with iD1 transmitter was used for the analysis. Each sample for analysis was prepared by mixing with KBr. IR spectra were recorded from 4000 cm^−1^ to 400 cm^−1^.

**Oligomerization process**

The oligomerization process was carried out on a chemical apparatus that included a glass tube with a septum and a magnetic stirrer with a heating function. The entire process was carried out under the pressure of an inert gas nitrogen and at atmospheric pressure. The reaction began by purging the glass tube of oxygen with nitrogen. Then 3 umol of the complex was dissolved in a mixture of deuterated toluene and anhydrous dimethylsulfoxide in a volume ratio of 1 mL:1 mL. Then 2.1 mL of methylaluminoxane and 3 mL of 2-chloro-2-propen-1-ol were added. For oligomerization of 2-propen-1-ol, 0.81 mL of activator was added followed by 3 mL of monomer. Both oligomerization processes were carried out for 45 min. In the case of the oligomerization of the chlorinated allyl alcohol derivative, a yellowish gel was obtained, while in the case of 2-propenol, a colorless product was obtained. Both products were washed with a mixture of 2M HCl and methanol (volume ratio of 1:1). The products were then filtered and dried for 48 h in an oven at 50 degrees Celsius.

**MALDI-TOF-MS analysis**

The oligomerization products were characterized by MALDI-TOF-MS (BrukerBiflex III spectrometer) using 2,5-dihydroxybenzoic acid as a matrix.

**Thermogravimetric analysis (TGA)**

Thermogravimetric analysis (TGA) was performed on a NETZSCH TG 209 instrument in the temperature range of 0 to 1000 °C in an argon atmosphere. The mass of samples subjected to thermal analysis was about 5 mg.

**Differential scanning calorimetry (DSC) analyses**

The differential scanning calorimetry (DSC) studies were performed using equipment from Mettler Toledo in a range from − 150 to 500 °C with a heating rate of 10 °C·min^−1^ in the inert atmosphere. The sample to the DSC measurements was about 5 mg. The calibration was carried out on the basis of standards for thermal analysis (indium, *n*-octane). Liquid nitrogen was used for cooling.

## 3. Results

### 3.1. X-ray Diffraction Structural Studies of Chromium and Cobalt Complexes

Designing the synthesis of new chemical compounds containing polycarboxylate ligands such as dipicolinate, nitrilotriacetate can be valuable for a number of reasons such as chemical properties, stability of complexes, physical properties, and practical applications. Polycarboxylate ligands have functional groups, such as carboxyl groups, that provide chemical properties such as the ability to form coordination chemical bonds with metals, which can lead to stable and unique compounds. The complexes containing polycarboxylate ligands tend to be more stable than those containing other ligands due to their ability to form coordination bonds with several metal atoms simultaneously, which increases their binding strength. Dipicolinate, nitrilotriacetate anions can affect the physical properties of the complexes, such as melting point, solubility, and stability in aqueous solutions. The polycarboxylate complexes are used in various applications such as catalytic chemical reactions, photocatalysis, and energy storage, and as functional and sensory materials. Designing the synthesis of new metal complexes containing polycarboxylate ligands can lead to stable, unique, and useful complexes with practical applications in various scientific and industrial fields.

#### 3.1.1. The Complex (**1**)

**Figure 1:**
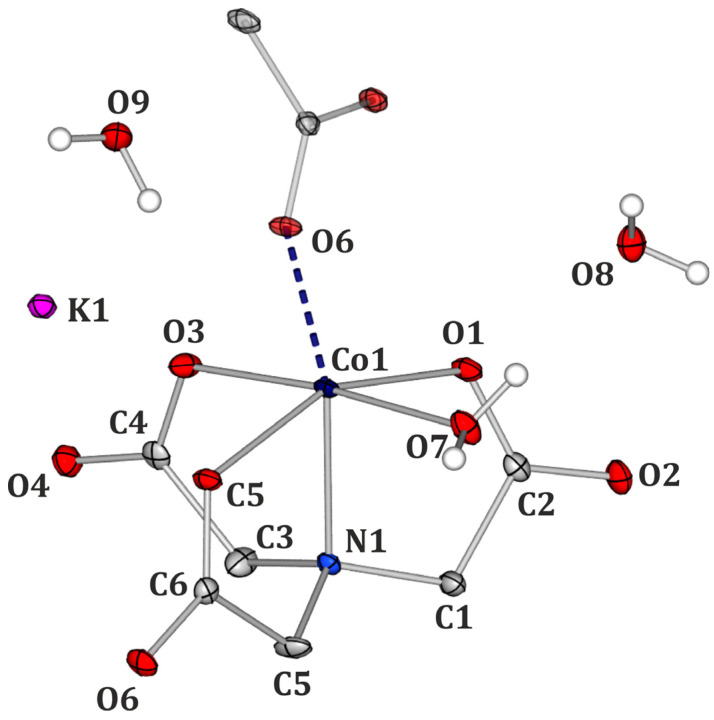
Molecular structure of the complex (**1**) including the labelling scheme. Atomic thermal motion is represented as ellipsoids (50% probability level); some hydrogen atom labels are omitted for clarity. Note that the cobalt atom is coordinated through O6 oxygen from the adjacent complex molecule (shown as semitransparent).

**Figure 2:**
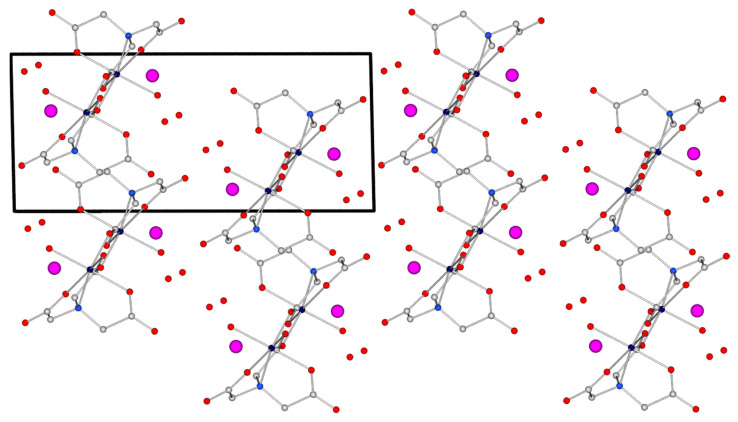
Packing of molecules of (**1**) along the *Y* axis. Hydrogen atoms are omitted for clarity. Note the layered architecture (large spheres are potassium cations and small red ones are oxygen atoms, respectively).

The crystal structure of this complex is already known, but it is described here for consistency with further results [38,39]. The complex (**1**) crystallizes in the monoclinic *P*2_1_/*n* space group with the [C_6_H_8_CoNO_7_]^−^ ion, potassium cation, and two water molecules comprising the asymmetric unit (ASU) (Figure 1). The fully deprotonated NTA chelating ligand coordinates to the cobalt cation through three oxygen atoms (O1, O3 & O5) and the nitrogen atom (N1). The remaining coordination sites of the metal center are saturated by the water molecule and the carboxyl group oxygen atom from the neighboring (1) species, yielding approximately octahedral geometry of the coordination sphere. Such linked adjacent anions form ‘zig-zag’-like chain motifs propagating along the [010] direction, which further interact one with another via C-H⋯O contacts (namely the C3-H3b⋯O6 and C5-H5b⋯O4 weak hydrogen bonds) leading to a layered supramolecular architecture. The resulting molecular layers are oriented parallel to the (001) crystal planes as shown in Figure 2. Water molecules and potassium ions interspace such layers, thus both contributing to the stabilization of molecular chains within the layer; they also ‘glue’ the neighboring layer motifs via strong electrostatic interactions of [C_6_H_8_CoNO_7_]^−^ with K^+^ anions and via hydrogen bonds formed by anionic species with water molecules.

#### 3.1.2. The Complex (**2**)

**Figure 3:**
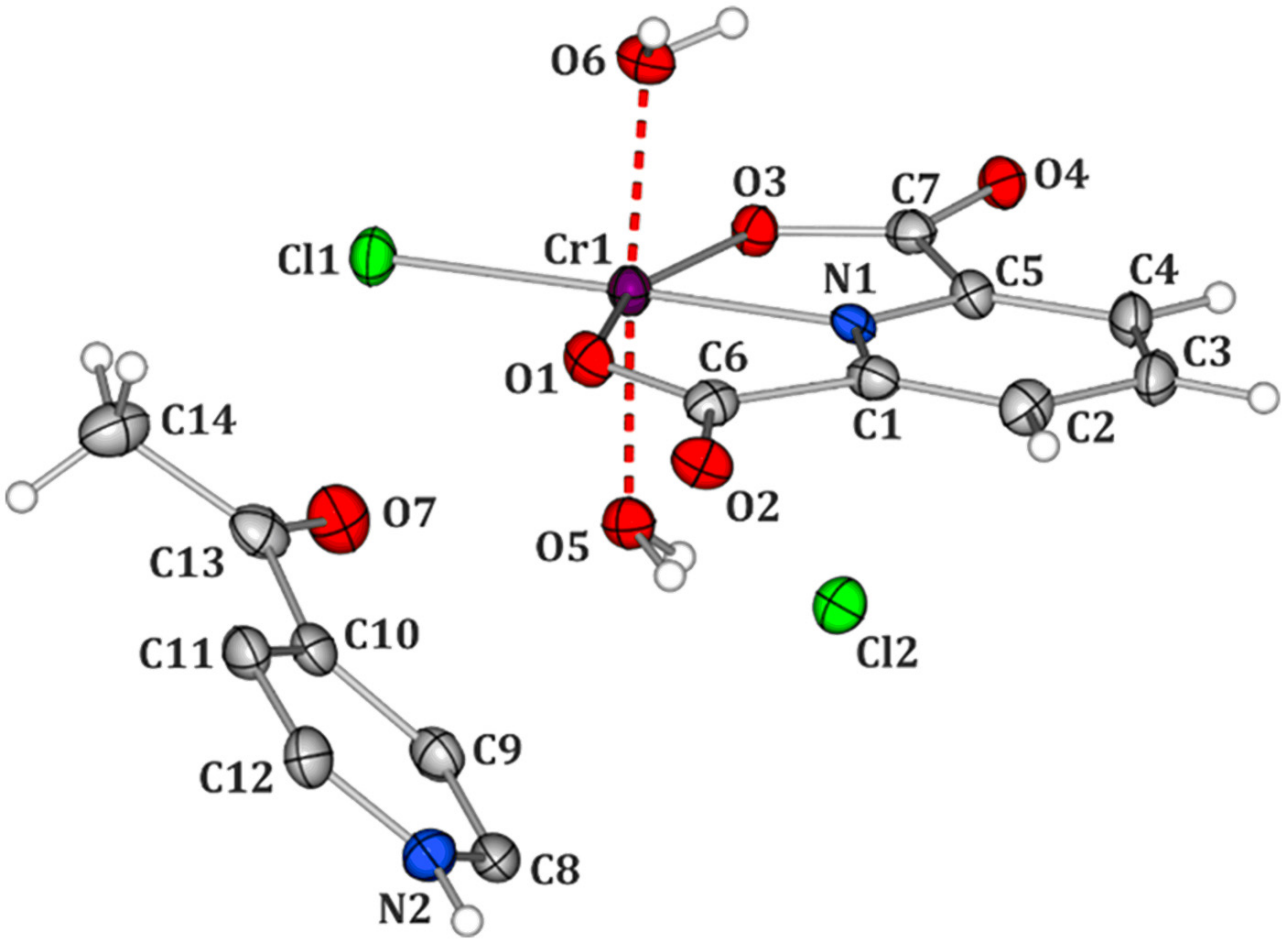
Molecular structure of the (**2**) complex with the labeling scheme depicted. Atomic thermal motion is represented as ellipsoids (50% probability level); some hydrogen atoms labels are omitted for clarity.

**Figure 4:**
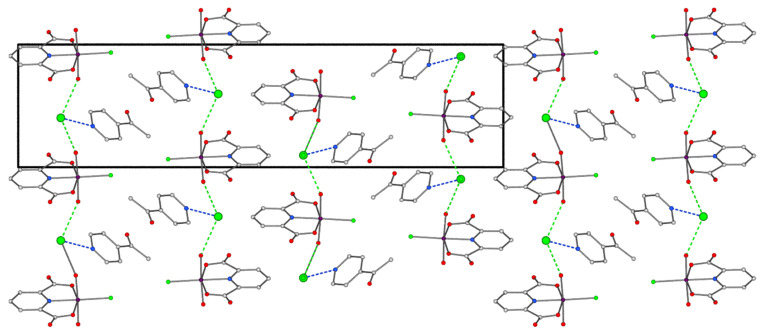
Packing of molecules of (**2**) along the *Z* axis. Hydrogen atoms are omitted for clarity. Note the layered architecture (larger green spheres are chloride anions).

The (**2**) compound crystallizes in the orthorhombic *P2_1_2_1_2_1_* space group with one complex molecule, C_7_H_7_ClCrNO_6_, the protonated 4-acetylpyridine species, and chloride anion in ASU (Figure 3). The dipic ligand coordinates to the chromium center via two oxygen atoms from the carboxylic groups (O1 & O3) and the N1 nitrogen atom. The approximately octahedral metal coordination sphere is completed by the chlorine ligand and two water molecules, located below and above the plane of the dipic ligand. The adjacent (**2**) species interact one with another through O-H⋯O hydrogen bonds (namely O6-H17⋯O2 and O5-H15⋯O4), which results in molecular chains along the [100] direction. The neighboring chains are connected together via the chloride counter ions, which also interact with the 4-acetylpyridine moieties (the hydrogen bonding data are included in the Supplementary Materials). This results in molecular layers parallel to the (100) crystal planes as shown in Figure 4. The structure is further stabilized mainly by some weaker contacts formed by the protons of the methyl groups and carbonyl oxygen atoms from the 4-acetylpyridine species, as well as by the chlorine ligand and aromatic ring fragments from the (**2**) moiety.

### 3.2. The Oligomerization Process of Olefins

**Scheme 1:**
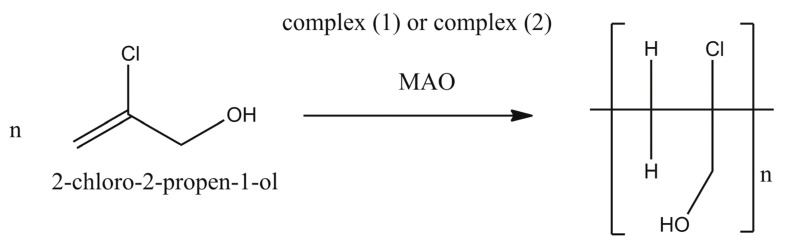
The oligomerization of 2-chloro-2-propen-1-ol in the presence of the complexes (**1**) or (**2**).

**Scheme 2:**
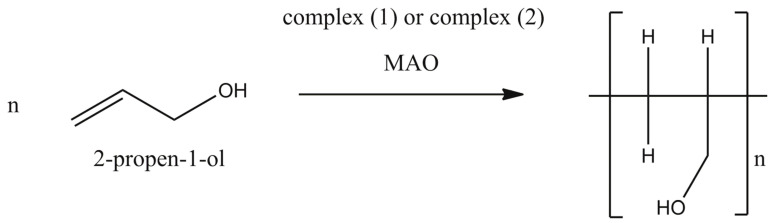
The oligomerization of 2-propen-1-ol in the presence of the complexes (**1**) or (**2**).

The oligomerizations of 2-chloro-2-propen-1-ol and 2-propen-1-ol proceeded according to the following reaction equation Scheme 1 and Scheme 2.

#### 3.2.1. The Complex (**1**) as a Precatalyst of the Oligomerization Process of Olefins

The nitrilotriacetate cobalt(II) complex was also used as a precatalyst in the oligomerization reactions of the chlorinated derivative of polyallyl alcohol and 2-propen-1-ol. Mixtures of the resulting oligomers were characterized by MALDI-TOF MS methods, from which the oligomeric chain lengths were determined. From the mass spectrum, we see the following peaks: *m*/*z* = 410.857 (5 mers), *m*/*z* = 588.85 (6 mers),*m*/*z*= 664.875 (7 mers), *m*/*z* = 842.857 (9 mers), *m*/*z* = 1020.828 (11 mer), 1198.793 (13 mer). The molecular peak was identified at *m*/*z* = 410.857. From the mass spectrum of the mixture of oligomeric chains of poly(2-propan-1ol), we see the following peaks: *m*/*z* = 354.799 (6 mers), *m*/*z* = 411.890 (7 mers), *m*/*z* = 478.156 (8 mers), 684.935 (11 mers), *m*/*z* = 916.740 (15 mers), 1078.745 (18 mers). The molecular peak was identified at *m*/*z* = 411.890.

**Figure 5:**
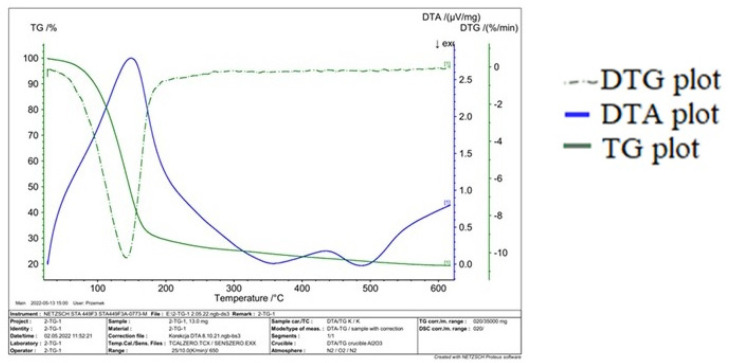
Thermal decomposition of the poly(2-chloro-2-propan-1-ol) sample.

The thermal stability of the oligomer mixtures was also investigated by thermogravimetric analysis. The sample of poly(2-chloro-2-propan-1-ol) oligomer mixture was characterized by rapid thermal decomposition in the range ∆T = 30–200 °C. Endothermic decomposition occurred at a rate of 10.27%/min. The residue after thermal decomposition (200 °C) was 29.45%, while the residue at T = 600 °C was 19.45% (Figure 5).

The onset of thermal decomposition of the poly(2-propan-1-ol) sample began at T = 30 °C; however, the range of rapid thermal decomposition of the sample was recorded at ∆T = 140–240 °C. Decomposition occurred at a rate of 17.70%/min, while the thermal decomposition residue was 30.77%, which underwent combustion in the temperature range ∆T = 240–320. The residue after combustion was 12.4%, while the residue at T = 600 °C was 1.25%.

A parameter often used to describe oligomers and polymers is the glass transition temperature. It is determined by differential scanning calorimetry. In the case of oligomers formed during oligomerization of 2-chloro-2-propen-1-ol, there are the following parameters describing the glass transition temperature: onset (−125.2), Mid (−120.6), inflection (−122.1), end (−117.4). On the spectrum obtained during DSC analysis, we also see a peak of 174.8 J/g at T = 109.2 °C.

For the DSC spectrum of the 2-propen-1-ol oligomer, the glass transition temperature was characterized by the following parameters: onset (−122.5), mid (−118.6), inflection (−118.6), end (−114.8). A peak of 573.2 J/g at T= 201.2 was also found.

#### 3.2.2. The Complex (**2**) as a Precatalyst of the Oligomerization Process of Olefins

The complex (**2**) was also used as a precatalyst in the oligomerization reactions of the chlorinated derivative of polyallyl alcohol and 2-propen-1-ol. Mixtures of the resulting oligomers were characterized by the MALDI-TOF MS methods, from which the oligomeric chain lengths were determined. From the mass spectrum, we see the following peaks: *m*/*z* = 100.588 (1 mer), *m*/*z* = 213.945 (2 mers), *m*/*z* = 370.993 (4 mers), *m*/*z* = 511,032 (5 mers), *m*/*z* = 665,035 (7 mers). The molecular peak was identified at *m*/*z* = 136,723. From the mass spectrum of the mixture of oligomeric chains of poly(2-propan-1ol), we see the following peaks: *m*/*z* = 136.741 (2 mers), *m*/*z* = 227.992 (3 mers), *m*/*z* = 689.013 (12 mers), *m*/*z* = 843.039 (14 mers), *m*/*z* = 1021.028 (17 mers), *m*/*z* = 1199.020 (20 mers), *m*/*z* = 1377.005 (23 mers). The molecular peak was identified at *m*/*z* = 136,741.

The sample underwent endothermic decomposition in a very wide temperature range ∆T = 40–320 °C. The speed of the main stage of thermal decomposition of the tested sample was 2.36%/min, so it was slower than in the case of a sample containing poly(2-propan-1-ol). Further, the decomposition residue at T = 600 °C was higher than in the case of the poly(2-propan-1-ol) sample because it was 41.2%.

The sample of 2-propen-1-ol underwent a clear endothermic decomposition in the temperature range of 150–255 °C. The speed of the thermal decomposition process was 35.35%/min. In the temperature range ∆T = 255–310 °C, the residue after the decomposition of the sample was burned, while the sample residue at the temperature T = 600 °C was 12.97% of the initial mass of the sample.

A parameter often used to describe oligomers and polymers is the glass transition temperature. It is determined by differential scanning calorimetry. In the case of oligomers formed during the oligomerization of 2-chloro-2-propen-1-ol there are the following parameters describing the glass transition temperature: onset (complex peak 1-61.9, complex peak 2-176.2), end (complex peak 1-150.6, complex peak 2-277.3), width (complex peak 1- 72.2, complex peak 2-75.8). On the spectrum obtained during DSC analysis, we also saw a peak of 239.4 J/g at T = 105.3 °C (complex peak 1) and of 88.34 J/g at T = 228.5 °C (complex peak 2).

**Figure 6:**
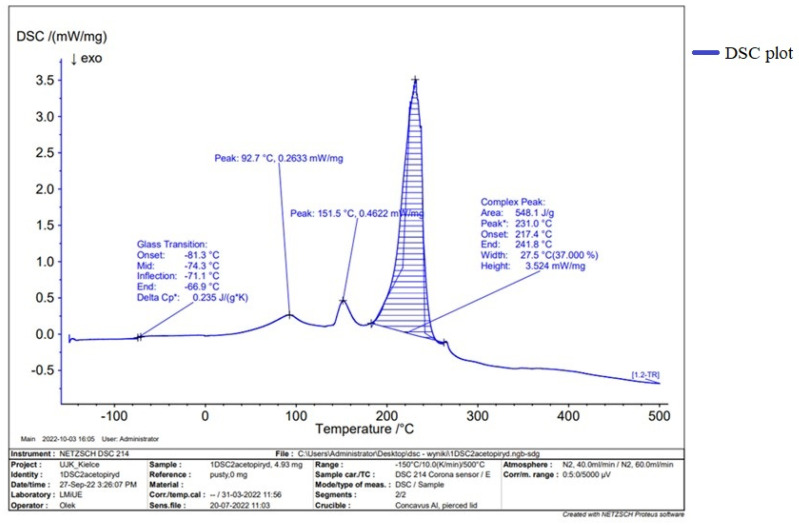
DSC analysis of the 2−propen−1−ol oligomers.

In the case of oligomers formed during the oligomerization of 2-propen-1-ol (Figure 6) there are the following parameters describing the glass transition temperature: onset (complex peak—217.4), end (complex peak—241.8), width (complex peak—27.5). On the spectrum obtained during DSC analysis, we also saw a peak of 548.1 J/g at T = 231.0 °C.

### 3.3. Catalytic Activity of (**1**) and (**2**) Complexes

The most important parameter characterizing a given precatalyst in oligomerization reactions is the catalytic activity. The catalytic activity for both oligomerization reactions was calculated on the basis of the following relationship:

catalytic activity = mp/(n·t)

where:

mp–the weight of oligomer sample [g]

n—amount of mmol of cobalt(II) or chromium(III) ions used in the oligomerization process [mmol]

t—time of the oligomerization process [h].

#### 3.3.1. The Complex (**1**)

The catalytic activity calculated on the basis of the abovementioned equation of the cobalt(II) complex was 972.45 g∙mmol^−1^∙h^−1^ in the oligomerization of 2-chloro-2-propen-1-ol and 1678.24 g∙mmol^−1^∙h^−1^ in the oligomerization of 2-propen-1-ol. Comparing the catalytic activity of the new precatalyst in the oligomerization reaction of 2-chloro-2-propen-1-ol with a precatalyst that was based on the same central cation, i.e., Co^2+^-[Co(ida)(H_2_O)_2_] (catalytic activity = 759.08 g∙mmol^−1^∙h^−1^) [40] or a precatalyst with the same anion derived from a tricarboxylic acid-nitrilotriacetate but a different central cation [Cr(NTA)(phen)]∙4H_2_O (catalytic activity = 213.92 g∙mmol^−1^∙h^−1^) [41], we can conclude that our new complex compound shows the highest catalytic activity.

#### 3.3.2. The Complex (**2**)

**Figure 7:**
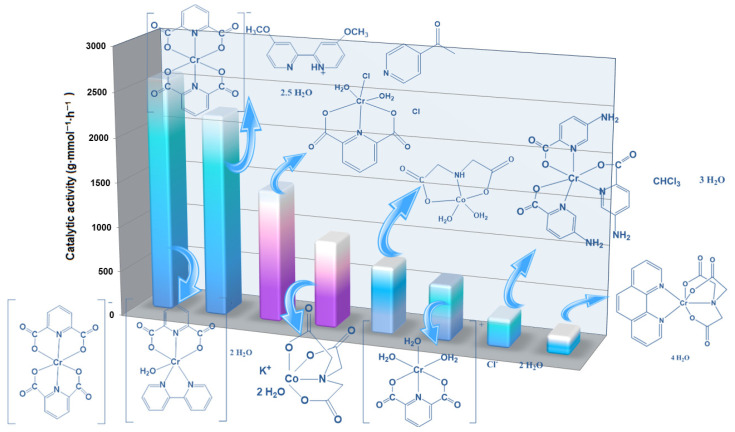
The catalytic activities of cobalt(II)-based and chromium(III)-based complexes in 2-chloro-2-propen-1-ol oligomerization (purple color indicates the activity for the complexes described in this report) [40,41,42,43,44].

The calculated catalytic activity of (**2**) was 1476.35 g∙mmol^−1^∙h^−1^ in the oligomerization of 2-chloro-2-propen-1-ol and 835.36 g∙mmol^−1^∙h^−1^ in the oligomerization of 2-propen-1-ol. As can be seen in Figure 7, the new chromium(III) and cobalt(II) complexes described in this paper against other chromium(III) and cobalt(II) complexes used as precatalysts in the oligomerization of 2-chloro-2-propen-1-ol are among the effective precatalysts showing very high catalytic activity.

The chromium(III) and cobalt(II) complexes described in this report are better precatalysts than other selected complexes catalyzing the oligomerization of olefins because they contain polycarboxylate ligands (multi-donor ligands) in the coordination sphere of chromium(III) cation and cobalt(II) cation.

Polycarboxylate complexes are very good precatalysts for olefin polymerization because of their catalytic properties and their ability to form active centers that catalyze olefin oligomerization and polymerization.

Polycarboxylate complexes with metal cations (such as cobalt or chromium) include those bound to polycarboxylates, which are organic ligands with chelating properties. Such complexes can act as Ziegler–Natty catalysts, meaning that their role is to activate monomer molecules by transferring electrons from the metal to the monomer molecule. The resulting active centers catalyze olefin oligomerization and polymerization.

Moreover, cobalt(II) and chromium(III) polycarboxylate complexes are also characterized by high activity, selectivity, and stability during polymerization reaction. They have the ability to polymerize various types of olefins, including ethylene, propylene, and butylene, and to form high-molecular-weight, high-quality polymers. As a result, polycarboxylate complexes are an important group of precatalysts in the polymer industry, and their catalytic properties are being intensively studied and developed for even more efficient and selective catalysts.

## 4. Conclusions

In summary, new complexes of chromium(III) and cobalt(II) were designed, synthesized, and described in detail, taking into account their structures. The complex (**1**) crystalized in the monoclinic *P*2_1_/*n* space group, whereas crystals of the (**2**) complex exhibited orthorhombic *P*2_1_2_1_2_1_ space group symmetry. Moreover, both complexes after activation were used to catalyze the allyl alcohol and 2-chloro-2-propen-1-ol oligomerizations. The catalytic activity was 972.45 g∙mmol^−1^∙h^−1^ for the cobalt(II)-based complex and 1678.24 g∙mmol^−1^∙h^−1^ for the 2-chloro-2-propen-1-ol and 2-propen-1-ol oligomerizations, respectively. In the case of the new chromium(III)-based complex compound, the values of catalytic activity were equal 1476.35 g∙mmol^−1^∙h^−1^ and 835.36 g∙mmol^−1^∙h^−1^ for the oligomerization of 2-chloro-2-propen-1-ol and the oligomerization of 2-propen-1-ol, respectively. Hence, it can be concluded that new complexes of chromium(III) and cobalt(II) are highly active precatalysts for the oligomerizations of selected olefins.

## Supplementary Materials

The following supporting information can be downloaded at: https://www.mdpi.com/article/10.3390/ma16093308/s1, Table S1. Selected X-ray data collection, processing and refinement parameters for (**1**) crystal structure. Table S2. Selected X-ray data collection, processing, and refinement parameters for (**2**) crystal structure. Figure S3. The mass spectrum of the 2-chloro-2-propen-1-ol oligomerization product ((**1**)). Figure S4. The mass spectrum of the 2-propen-1-ol oligomerization product ((**1**)). Figure S5. Thermal decomposition of the poly(2-propan-1-ol) sample ((**1**)). Figure S6. DSC analysis of the 2-chloro-2-propen-1-ol oligomers ((**1**)). Figure S7. DSC analysis of the 2-propen-1-ol oligomers ((**1**)). Figure S8. The mass spectrum of the 2-chloro-2-propen-1-ol oligomerization product ((**2**)). Figure S9. The mass spectrum of the 2-propen-1-ol oligomerization product ((**2**)). Figure S10. Thermal decomposition of the poly(2-chloro-2-propan-1-ol) sample ((**2**)). Figure S11. Thermal decomposition of the poly(2-propan-1-ol) sample ((**2**)). Figure S12. DSC analysis of the 2-chloro-2-propen-1-ol oligomers ((**2**)).
