# Supplementary material for: Catalytic Properties of Two Complexes of chromium(III) and cobalt(II) with Nitrilotriacetate, Dipicolinate, and 4-Acetylpyridine
Source: Materials (Basel). 2023 Apr 23;16(9):3308. doi: 10.3390/ma16093308 (PMC10179121; doi:10.3390/ma16093308)
Supplement: Supplementary file 1 [file materials-16-03308-s001.zip › materials-2289530-supplementary.pdf]

materials

Supplementary Materials

Catalytic Properties of Two Complexes of chromium(III) and cobalt(II) with Nitrilotriacetate, Dipicolinate, and 4-Acetylpyridine

Jacek Malinowski [1], Joanna Drzeżdżon [1,*], Katarzyna N. Jarzembska [2], Radosław Kamiński [2], Przemysław Rybiński [3], Barbara Gawdzik [3] and Dagmara Jacewicz [1]

[1] Faculty of Chemistry, University of Gdansk (Gdańsk), Wita Stwosza 63, 80-308 Gdansk, Poland
[2] Department of Chemistry, University of Warsaw, Żwirki i Wigury 101, 02-089 Warsaw, Poland
[3] Institute of Chemistry, Jan Kochanowski University, Uniwersytecka 7, 25-406 Kielce, Poland
* Correspondence: joanna.drzezdzon@ug.edu.pl

Table S1. Selected X-ray data collection, processing and refinement parameters for crystal structure **(1)**.

Moiety formula	$[C_6H_8CoNO_7]^-$, K^+, 2 H_2O
Moiety formula mass, M_r / a.u.	340.2
Crystal system	monoclinic
Space group	$P2_1/n$ (No. 14)
Z	4
F_{000}	684
Crystal color & shape	pink plate
Crystal size / mm³	0.04×0.12×0.18
T / K	100
a / Å	7.095(2)
b / Å	9.974(2)
c / Å	16.223(3)
α / Å	90
β / Å	91.21(3)
γ / Å	90
V / Å³	1147.7(5)
d_{calc} / g·cm⁻³	1.948
θ range	5.21°–76.09°
Absorption coefficient, μ / mm⁻¹	15.424
No. of reflections collected / unique	14542 / 2394
R_{int}	5.89%
No. of reflections with $I > 3\sigma(I)$	2245
No. of parameters / restraints / constraints	181 / 6 / 30
$R[F]$ ($I > 3\sigma(I)$)	2.70%
$R[F]$ (all data)	2.93%
$\varrho_{\mathrm{res}}^{\mathrm{min/max}}$ / e·Å⁻³	−0.34 / +0.36
CCDC code	2116382

Table S2. Selected X-ray data collection, processing and refinement parameters for crystal structure **(2)**.

Moiety formula	$C_7H_7ClCrNO_6$, $[C_7H_8NO]^+$, Cl^-
Moiety formula mass, M_r / a.u.	446.18
Crystal system	orthorhombic
Space group	$P2_12_12_1$ (No. 19)
Z	4
F_{000}	908
Crystal color & shape	blue-green cut crystal
Crystal size / mm^3	0.03×0.06×0.09
T / K	100
a / Å	33.623(7)
b / Å	8.511(2)
c / Å	6.099(2)
α / Å	90
β / Å	90
γ / Å	90
V / Å^3	1745.4(8)
d_{calc} / g·cm^{-3}	1.698
θ range	2.63°–76.23°
Absorption coefficient, μ / mm^{-1}	8.599
No. of reflections collected / unique	23856 / 3647
R_{int}	11.08%
No. of reflections with $I > 3\sigma(I)$	2975
No. of parameters / restraints / constraints	247 / 4 / 48
$R[F]$ ($I > 3\sigma(I)$)	4.33%
$R[F]$ (all data)	5.74%
$\varrho_{res}^{min/max}$ / e·Å^{-3}	−0.42 / +0.42
CCDC code	2116383

Table S3. Selected hydrogen bonds (HBs) present in the crystal structure of **(1)**. Abbreviations: *d* – distance, *θ* – angle, D – HB donor, A – HB acceptor. Symmetry transformations are omitted for clarity (they are present in the CIF files).

Interaction	$d_{D–H}$ / Å	$d_{H\cdots A}$ / Å	$d_{D\cdots A}$ / Å	$\theta_{D–H\cdots A}$ / °
O7–H6⋯O8	0.82(2)	1.972(19)	2.777(2)	167(2)
O7–H7⋯O9	0.82(2)	1.885(18)	2.689(2)	167(2)
O8–H8⋯O4	0.82(1)	1.928(15)	2.745(2)	174(3)
O8–H9⋯O2	0.820(3)	2.147(10)	2.927(3)	159(3)
O9–H10⋯O2	0.82(2)	2.00(2)	2.823(2)	178(2)
O9–H11⋯O3	0.82(2)	1.99(2)	2.785(2)	164(2)

Table S4. Selected hydrogen bonds (HBs) present in the crystal structure of **(2)**. Abbreviations: *d* – distance, *θ* – angle, D – HB donor, A – HB acceptor. Symmetry transformations are omitted for clarity (they are present in the CIF files).

Interaction	$d_{D–H}$ / Å	$d_{H\cdots A}$ / Å	$d_{D\cdots A}$ / Å	$\theta_{D–H\cdots A}$ / °
O5–H15⋯O4	0.82(4)	1.87(4)	2.689(5)	172(6)
O5–H16⋯Cl2	0.82(4)	2.22(4)	3.031(3)	169(5)
O6–H17⋯O2	0.82(4)	1.93(4)	2.744(5)	171(5)
O6–H18⋯Cl2	0.82(3)	2.23(3)	3.033(4)	168(5)
N2–H19⋯Cl2	0.87	2.36	3.192(4)	160.93

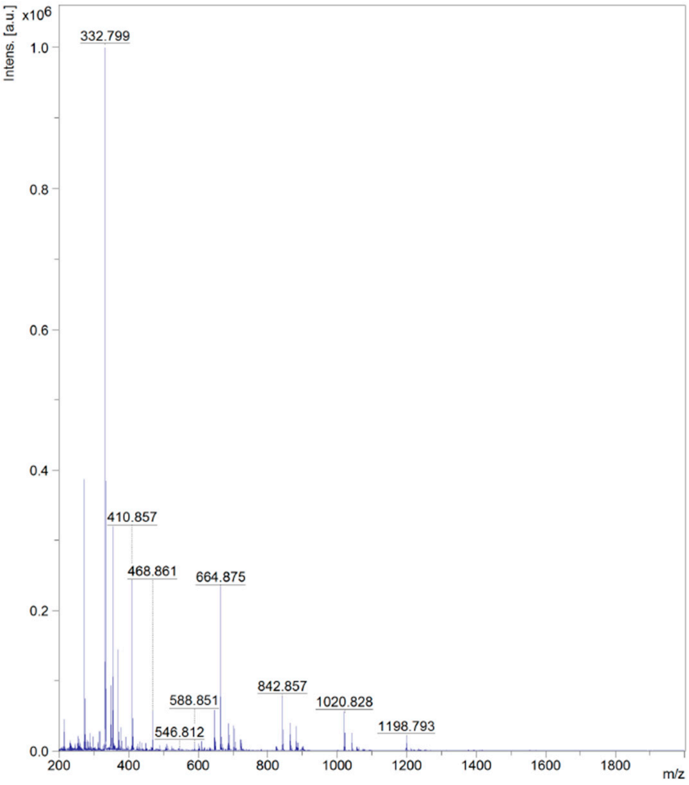

Figure S1. The mass spectrum of the 2-chloro-2-propen-1-ol oligomerization product (**CoNTA)**.

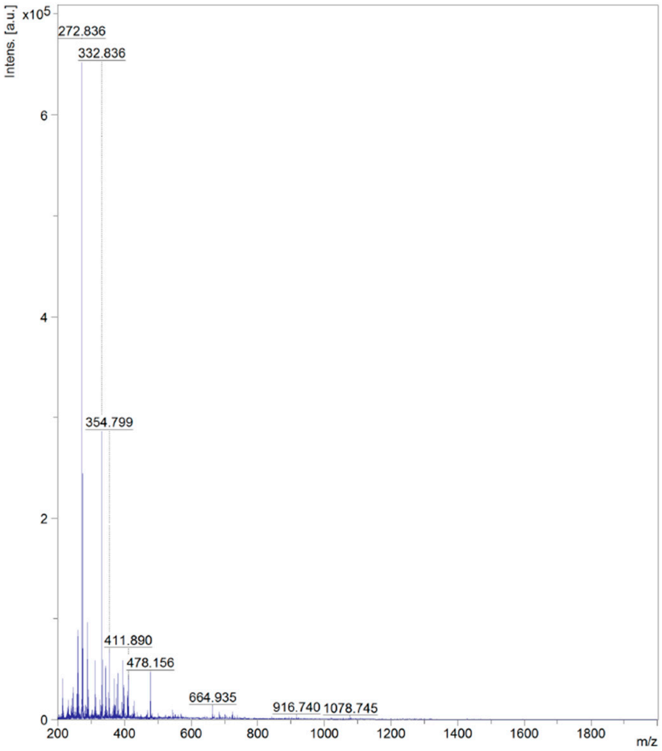

Figure S2. The mass spectrum of the 2-propen-1-ol oligomerization product (**CoNTA)**.

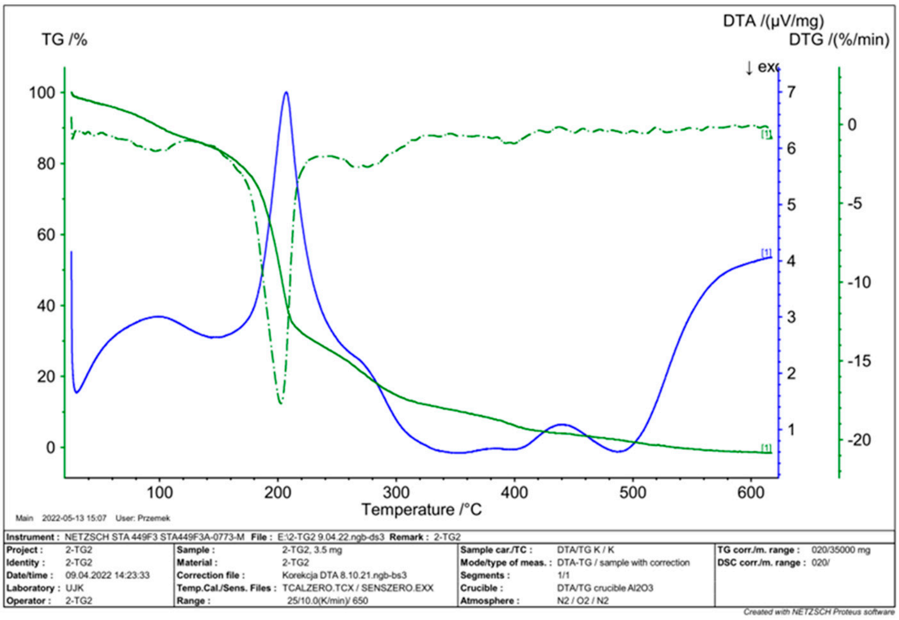

Figure S3. Thermal decomposition of the poly(2-propan-1-ol) sample (**CoNTA)**.

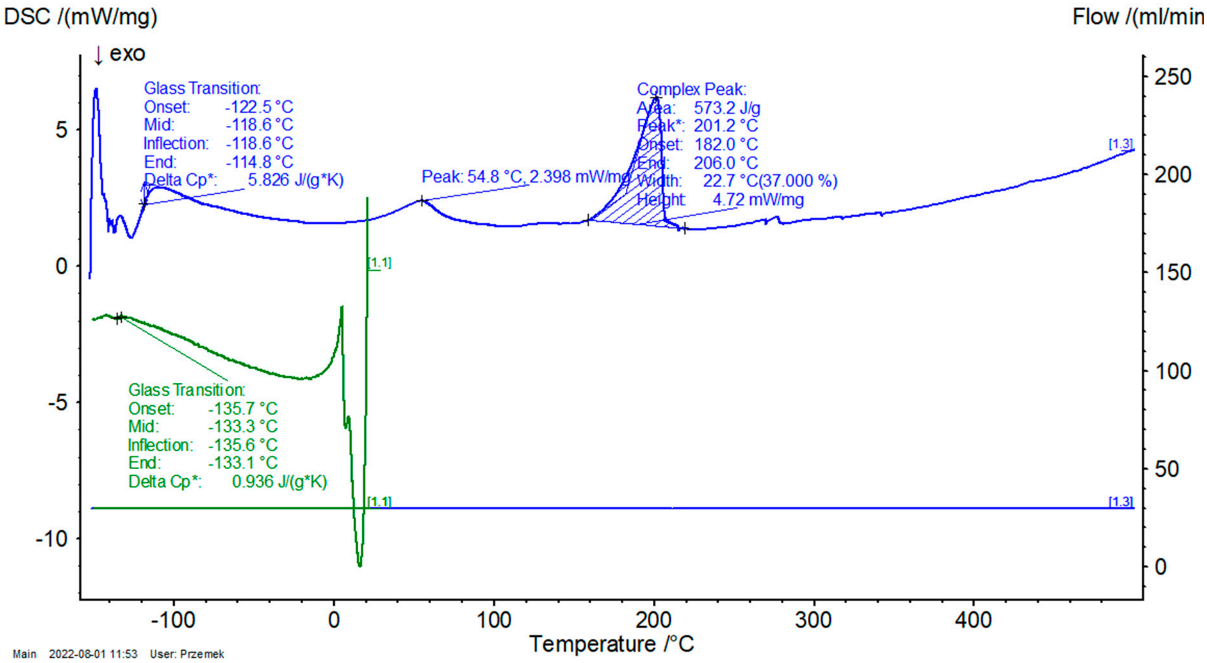

Figure S4. DSC analysis of the 2-chloro-2-propen-1-ol oligomers (**CoNTA)**.

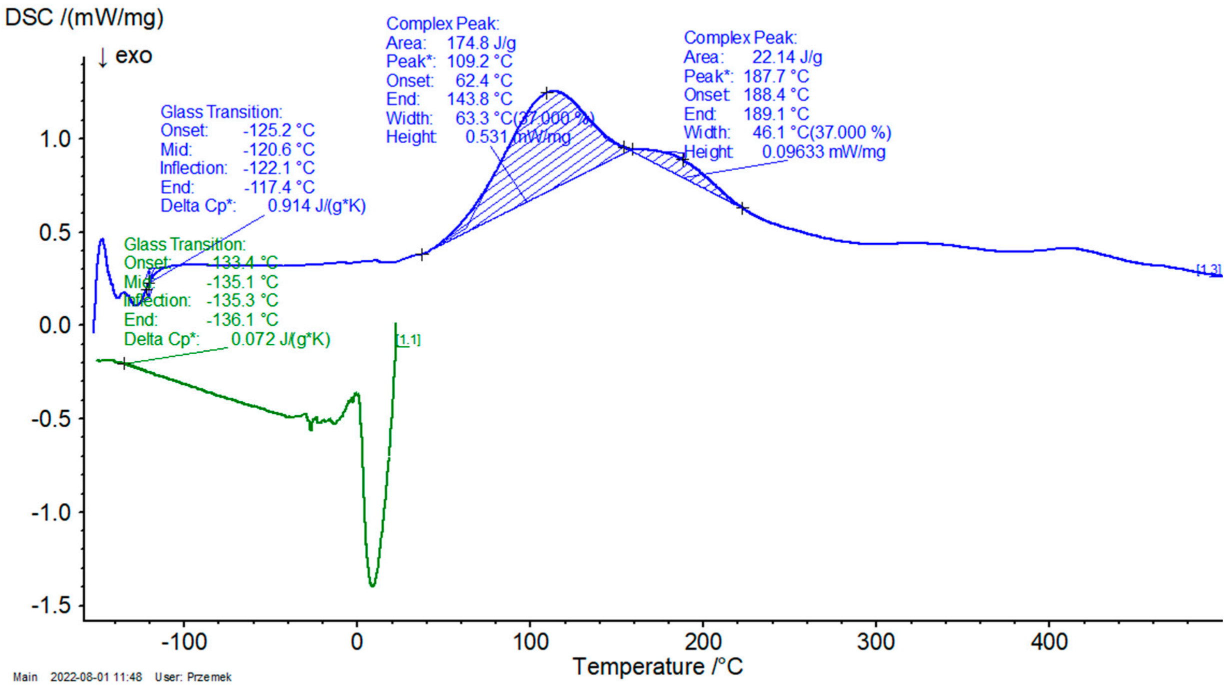

Figure S5. DSC analysis of the 2-propen-1-ol oligomers (**CoNTA)**.

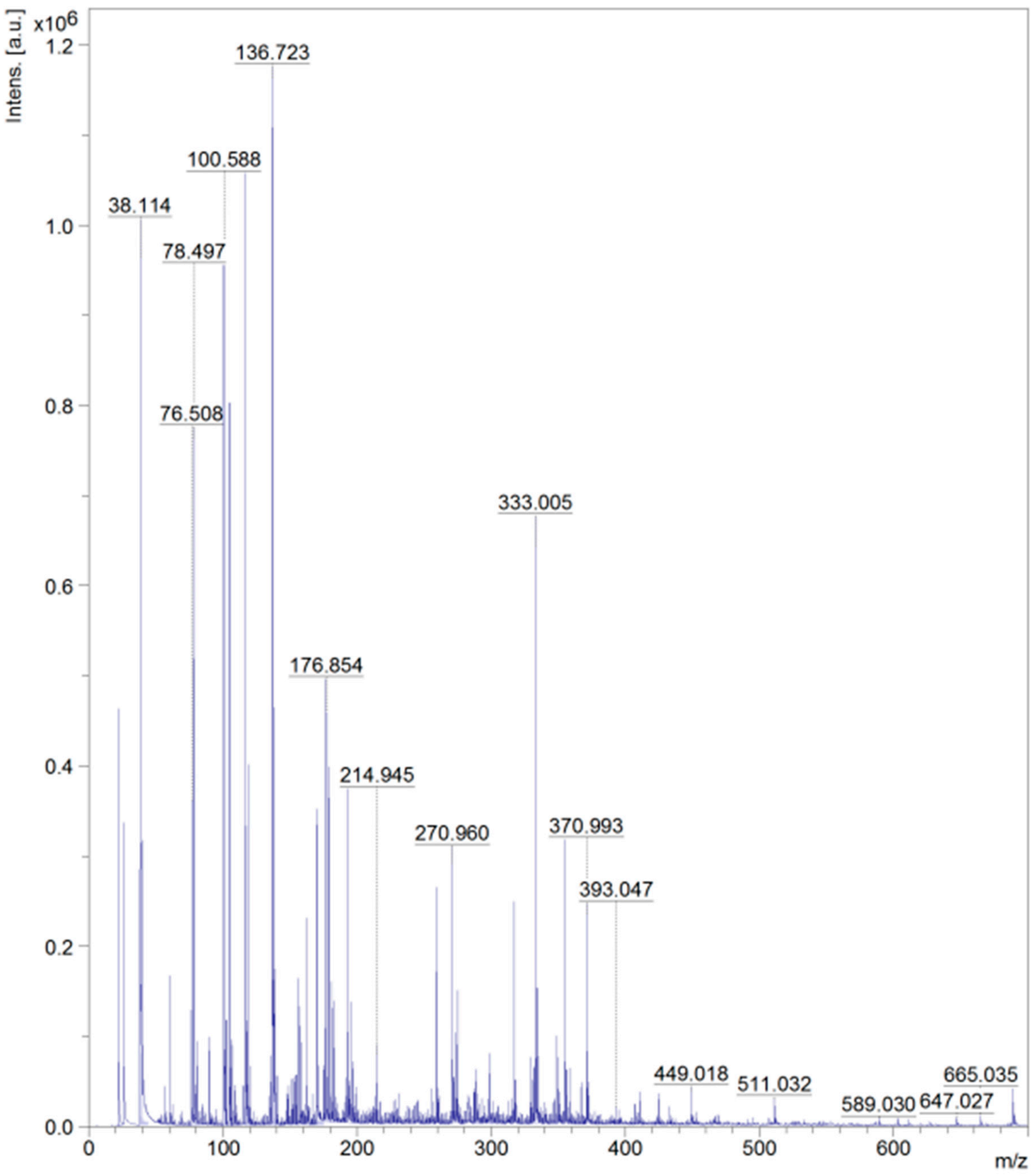

Figure S6. The mass spectrum of the 2-chloro-2-propen-1-ol oligomerization product (**Crdipic4acetyl)**.

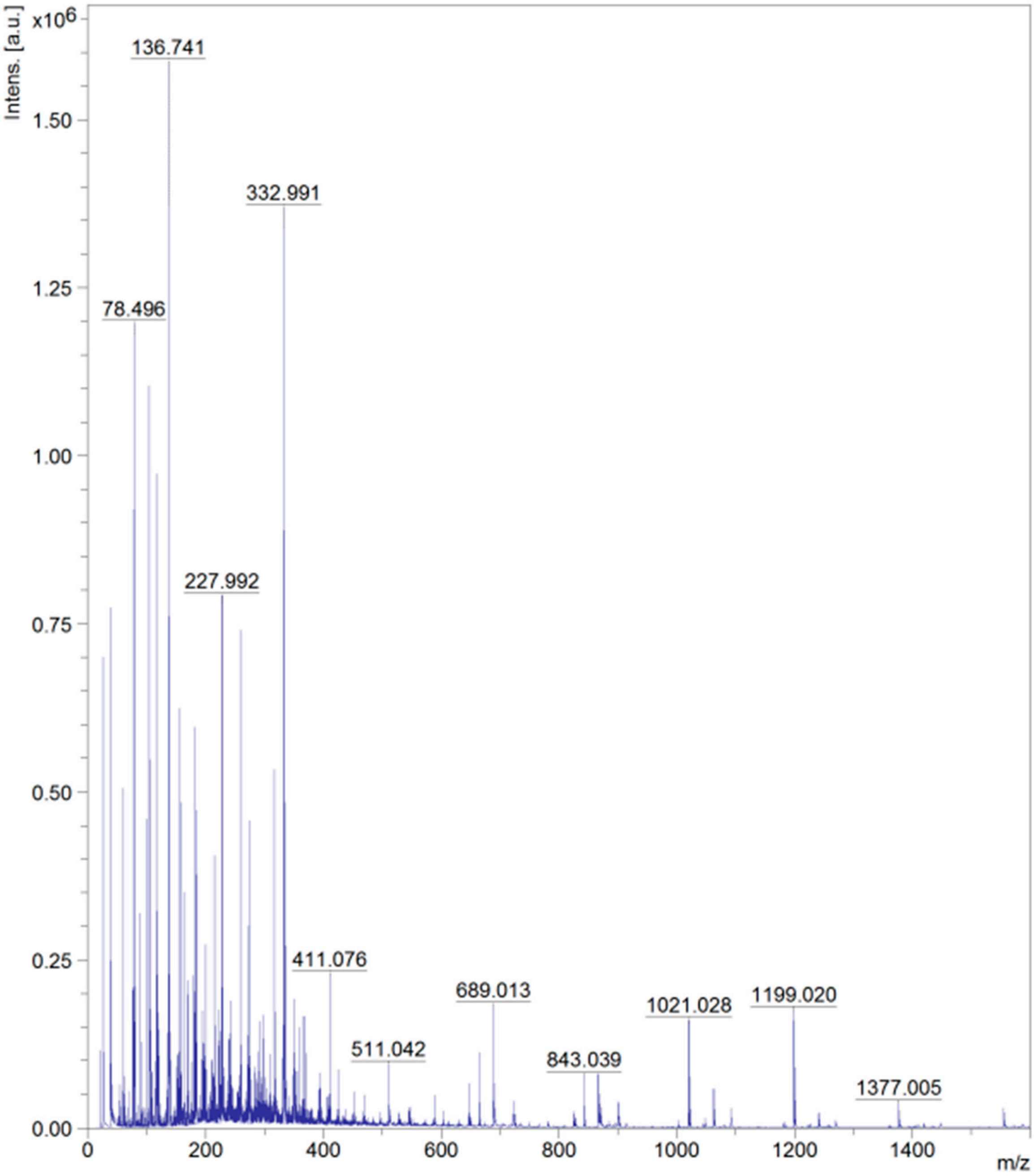

Figure S7. The mass spectrum of the 2-propen-1-ol oligomerization product (**Crdipic4acetyl)**.

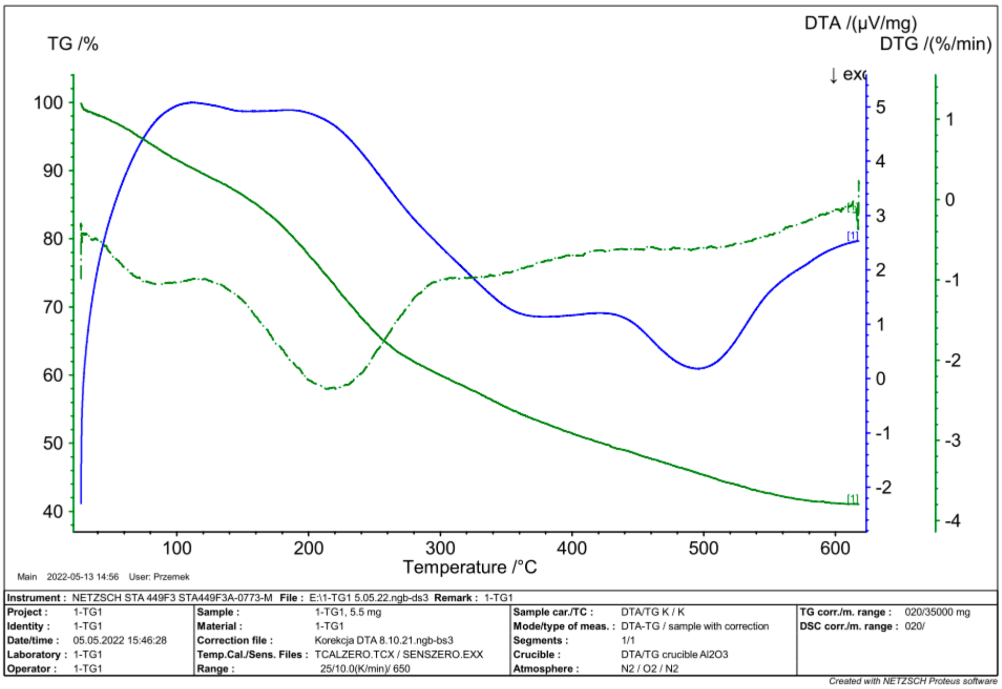

Figure S8. Thermal decomposition of the poly(2-chloro-2-propan-1-ol) sample (**Crdipic4acetyl)**.

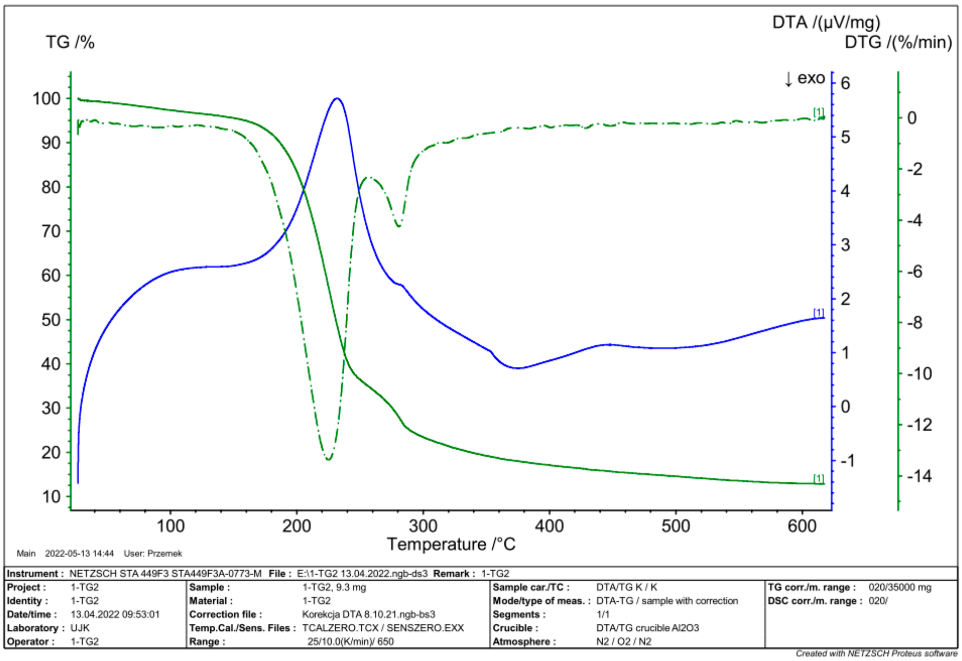

Figure S9. Thermal decomposition of the poly(2-propan-1-ol) sample (**Crdipic4acetyl)**.

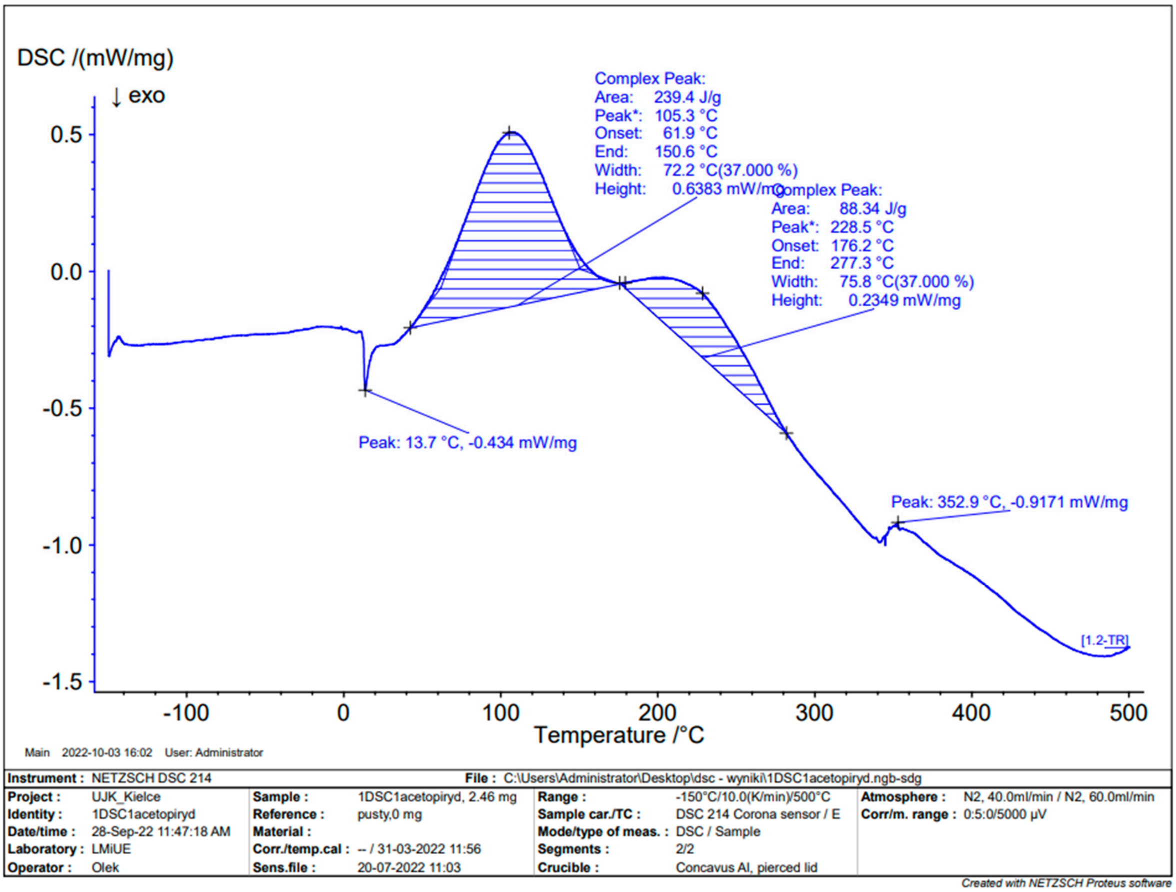

Figure S10. DSC analysis of the 2-chloro-2-propen-1-ol oligomers (**Crdipic4acetyl)**.
